# The CARBA-MAP study: national mapping of carbapenemases in Spain (2014–2018)

**DOI:** 10.3389/fmicb.2023.1247804

**Published:** 2023-09-08

**Authors:** Irene Gracia-Ahufinger, Laura López-González, Francisco José Vasallo, Alicia Galar, María Siller, Cristina Pitart, Iván Bloise, Miriam Torrecillas, Desirée Gijón-Cordero, Belén Viñado, Javier Castillo-García, Rainer Campo, Xavier Mulet, Ana Madueño-Alonso, Francisco Javier Chamizo-López, Maitane Arrastia-Erviti, Fátima Galán-Sánchez, Melisa Fernández-Quejo, Juan Carlos Rodríguez-Díaz, María Nieves Gutiérrez-Zufiaurre, Manuel Angel Rodríguez-Maresca, María del Pilar Ortega-Lafont, Genoveva Yagüe-Guirao, Lucía Chaves-Blanco, Javier Colomina-Rodríguez, María Reyes Vidal-Acuña, María Eugenia Portillo, Francisco Franco-Álvarez de Luna, María José Centelles-Serrano, José Manuel Azcona-Gutiérrez, Alberto Delgado-Iribarren García Campero, Sonia Rey-Cao, Patricia Muñoz, Jorge Calvo-Montes, Yuliya Zboromyrska, David Grandioso, Jordi Càmara, Rafael Cantón, Nieves Larrosa-Escartín, Jazmín Díaz-Regañón, Luis Martínez-Martínez

**Affiliations:** ^1^Unit of Microbiology, Reina Sofia University Hospital, Cordoba, Spain; ^2^Maimonides Biomedical Research Institute of Cordoba (IMIBIC), Cordoba, Spain; ^3^Center for Biomedical Research in Infectious Diseases (CIBERINFEC), Carlos III Health Institute (ISCIII), Madrid, Spain; ^4^Clinical Microbiology Service, IML, San Carlos Clinical University Hospital, Madrid, Spain; ^5^Health Research Institute of the Hospital Clínico San Carlos (IdISSC), Madrid, Spain; ^6^Microbiology Service, Vigo University Hospital Complex (CHUVI), Vigo, Spain; ^7^Health Research Institute Galicia Sur (IISGS), Vigo, Spain; ^8^Clinical Microbiology and Infectious Diseases Department, Hospital General Universitario Gregorio Marañón, Madrid, Spain; ^9^Health Research Institute Hospital Gregorio Marañón, Madrid, Spain; ^10^Medicine Department, School of Medicine, Universidad Complutense de Madrid, Madrid, Spain; ^11^CIBER de Enfermedades Respiratorias (CIBERes), Instituto de Salud Carlos III, Madrid, Spain; ^12^Microbiology Service, Marqués de Valdecilla University Hospital, Santander, Spain; ^13^Marqués de Valdecilla Health Research Institute (IDIVAL), Santander, Spain; ^14^Microbiology Service, Hospital Clinic, Barcelona, Spain; ^15^Department of Basic Clinical Practice, University of Barcelona, Barcelona, Spain; ^16^Institute of Global Health of Barcelona, Barcelona, Spain; ^17^Clinical Microbiology Department, La Paz University Hospital, Madrid, Spain; ^18^Hospital La Paz Institute for Health Research (IdiPaz), Madrid, Spain; ^19^Clinical Microbiology Department, Bellvitge University Hospital, L'Hospitalet de Llobregat, Spain; ^20^Microbiology Service, Ramón y Cajal University Hospital, Madrid, Spain; ^21^Ramón y Cajal Institute for Health Research (IRYCIS), Madrid, Spain; ^22^Microbiology Service, Vall d'Hebron University Hospital, Barcelona, Spain; ^23^Vall d'Hebron Research Institute (VHIR), Barcelona, Spain; ^24^Microbiology Service, Lozano Blesa Clinical University Hospital, Zaragoza, Spain; ^25^Institute for Health Research Aragón (IIS Aragón), Zaragoza, Spain; ^26^School of Medicine, University of Zaragoza, Zaragoza, Spain; ^27^Microbiology Service, Asturias Central University Hospital, Oviedo, Spain; ^28^Microbiology Service, Son Espases University Hospital, Palma de Mallorca, Spain; ^29^Institute for Health Research Illes Balears (IdISBa), Palma, Spain; ^30^Microbiology Service, University Hospital of the Canary Islands, Tenerife, Spain; ^31^Microbiology Service, Doctor Negrín University Hospital of Gran Canaria, Gran Canaria, Spain; ^32^Microbiology Service, University Hospital of Donostia, San Sebastián, Spain; ^33^Microbiology Service, Puerta del Mar University Hospital, Cádiz, Spain; ^34^Microbiology Service, A Coruña University Hospital, A Coruña, Spain; ^35^Microbiology Service, General University Hospital Dr. Balmis, Alicante, Spain; ^36^Health and Biomedical Research Institute of Alicante (ISABIAL), Alicante, Spain; ^37^Microbiology Service, University Hospital of Salamanca, Salamanca, Spain; ^38^Laboratory Unit, Microbiology Section, Torrecardenas University Hospital, Almería, Spain; ^39^Microbiology Service, University Hospital of Burgos, Burgos, Spain; ^40^Virgen de la Arrixaca University Hospital, Murcia, Spain; ^41^Department of Genetics and Microbiology, University of Murcia, Murcia, Spain; ^42^Murcian Institute for Biomedical Research (IMIB), Murcia, Spain; ^43^Microbiology Service, San Cecilio Clinical University Hospital, Granada, Spain; ^44^Microbiology Service, Hospital Clínico Universitario de Valencia, Valencia, Spain; ^45^Microbiology Service, Cáceres University Hospital Complex, Cáceres, Spain; ^46^Clinical Microbiology Service, University Hospital of Navarra, Pamplona, Spain; ^47^Health Research Institute of Navarra (IdiSNA), Pamplona, Spain; ^48^Microbiology Service, Hospital Juan Ramón Jiménez, Huelva, Spain; ^49^Microbiology Area, Clinical Laboratory, Hospital of Tortosa Virgen de la Cinta, Tortosa, Spain; ^50^Institute for Health Research Pere Virgili, Tortosa, Spain; ^51^Microbiology Laboratory, San Pedro Hospital, Logroño, Spain; ^52^Institut Investigacio Biomedica de Bellvitge (IDIBELL), L'Hospitalet de Llobregat, Spain; ^53^Medical Department, MSD España, Madrid, Spain; ^54^Department of Agricultural Chemistry, Soil Science and Microbiology, University of Cordoba, Cordoba, Spain

**Keywords:** *Klebsiella pneumoniae*, *Escherichia coli*, *Enterobacter cloacae* complex, *Klebsiella* (*Enterobacter*) *aerogenes*, *Pseudomonas aeruginosa*, carbapenemases, geographical distribution

## Abstract

**Introduction:**

Infections caused by carbapenem-resistant Enterobacterales (CRE) and carbapenem-resistant *Pseudomonas aeruginosa*, including isolates producing acquired carbapenemases, constitute a prevalent health problem worldwide. The primary objective of this study was to determine the distribution of the different carbapenemases among carbapenemase-producing Enterobacterales (CPE, specifically *Escherichia coli*, *Klebsiella pneumoniae*, *Enterobacter cloacae* complex, and *Klebsiella aerogenes*) and carbapenemase-producing *P. aeruginosa* (CPPA) in Spain from January 2014 to December 2018.

**Methods:**

A national, retrospective, cross-sectional multicenter study was performed. The study included the first isolate per patient and year obtained from clinical samples and obtained for diagnosis of infection in hospitalized patients. A structured questionnaire was completed by the participating centers using the REDCap platform, and results were analyzed using IBM SPSS Statistics 29.0.0.

**Results:**

A total of 2,704 carbapenemase-producing microorganisms were included, for which the type of carbapenemase was determined in 2692 cases: 2280 CPE (84.7%) and 412 CPPA (15.3%), most often using molecular methods and immunochromatographic assays. Globally, the most frequent types of carbapenemase in Enterobacterales and *P. aeruginosa* were OXA-48-like, alone or in combination with other enzymes (1,523 cases, 66.8%) and VIM (365 cases, 88.6%), respectively. Among Enterobacterales, carbapenemase-producing *K. pneumoniae* was reported in 1821 cases (79.9%), followed by *E. cloacae* complex in 334 cases (14.6%). In Enterobacterales, KPC is mainly present in the South and South-East regions of Spain and OXA-48-like in the rest of the country. Regarding *P. aeruginosa*, VIM is widely distributed all over the country. Globally, an increasing percentage of OXA-48-like enzymes was observed from 2014 to 2017. KPC enzymes were more frequent in 2017–2018 compared to 2014–2016.

**Discussion:**

Data from this study help to understand the situation and evolution of the main species of CPE and CPPA in Spain, with practical implications for control and optimal treatment of infections caused by these multi-drug resistant organisms.

## Introduction

Multiple antibiotic resistance has emerged as major public health threat. Infections caused by antibiotic-resistant bacteria are associated with significant morbidity and mortality (Prestinaci et al., 2015; Li et al., 2022). Currently the major problem is caused by multi-drug resistant (MDR) Gram-negative bacteria, particularly Enterobacterales, *Pseudomonas aeruginosa* or *Acinetobacter baumannii* presenting resistance to carbapenems (Sheu et al., 2019; Jean et al., 2022; Tenover et al., 2022). All these microorganisms are included in the WHO list of priority pathogens that urgently require investigation and development of new and effective antibiotic treatments (Tacconelli et al., 2018). For years, multidrug resistance in Enterobacterales has been related to the production of extended-spectrum β-lactamases (ESBLs), for which carbapenems have been considered first-line therapeutic options. However, their use has led to a more serious problem: the emergence of carbapenem-resistant Enterobacterales (CRE; Paño Pardo et al., 2014; van Duin and Doi, 2017; Suay-Garcia and Perez-Gracia, 2019). Similarly, there has been an increasing prevalence of infections produced by MDR and extensively drug-resistant (XDR) *P. aeruginosa*, which results from the extraordinary ability of this organism to develop resistance to nearly all available antibiotics through the selection of mutations in chromosomal genes or the acquisition of mobile genes (Del Barrio-Tofiño et al., 2019; Horcajada et al., 2019).

The principal mechanism of carbapenem resistance in Enterobacterales is the production of β-lactamases, particularly carbapenemases (Martínez-Martínez and González-López, 2014; Lepe and Martínez-Martínez, 2022), from Ambler Class A [e.g., KPC (*Klebsiella pneumoniae* carbapenemase)], Class B metallo-β-lactamases [MBL; e.g., NDM (New Delhi metallo-β-lactamase), VIM (Verona integron-encoded metallo-β-lactamase), IMP (Imipenemase)] and Class D [e.g., OXA-48-like (oxacillinase)], and rarely identified in clinical strains Class C carbapenemases (e.g., CMY-10, ACT-28; Martínez-Martínez and González-López, 2014; Bush, 2018; Tooke et al., 2019). Additionally, altered permeability (caused by loss or structural alterations of porins) and, possibly, overproduction of efflux pump(s) modulates the final level of resistance (Martínez-Martínez, 2008; Ferrand et al., 2020).

In 2015, the European Centre for Disease Prevention and Control (ECDC), evaluated the evolving epidemiology of carbapenemase-producing Enterobacterales (CPE) in 38 EU countries and documented a worsening epidemiological situation, primarily due to the rapid spread of OXA-48-like and NDM-producing organisms (Albiger et al., 2015). Another survey in 37 European countries in 2018 reported an ongoing dissemination of CPE over the past years in Europe (Brolund et al., 2019). In Spain, a multicenter study on Enterobacterales conducted in 2009 in 35 hospitals assessing the prevalence of plasmid-mediated AmpC (pAmpC) and carbapenemases found that only 0.04% of the evaluated organisms produced carbapenemases (most frequently VIM-1 and IMP-22; Miro et al., 2013). Four years later, the prevalence of carbapenemases in Spanish centres had increased to 1.7% in *K. pneumoniae* and 0.3% in *E. coli*, with a predominance of OXA-48-like and VIM enzymes, broadly distributed throughout the country, and KPC variants causing significant outbreaks in some hospitals (Oteo et al., 2015). Moreover, The EuSCAPE study performed in 2016 showed an increase in KPC to 7.8% and a decrease in OXA-48 to 69.8% among CRE in Spain compared to previous Spanish studies (Grundmann et al., 2017). In the recent CARB-ES-19 multicenter study (71 hospitals) evaluating 403 isolates, including carbapenemase-producing *K. pneumoniae* and *E. coli* (February–may 2019), the main carbapenemase genes identified in *K. pneumoniae* were *bla*OXA-48 (69.8%) and *bla*KPC-3 (16.7%) related to clones ST307/OXA-48 (16.4%), ST11/OXA-48 (16.4%), and ST512-ST258/KPC (13.8%; Cañada-García et al., 2022).

Less information is available regarding CRE other than *K. pneumoniae* and *E. coli*. Multiple carbapenemases have been described in both *E. cloacae* and *K. (E.) aerogenes* but, in Europe, the most common enzymes are of VIM and OXA-48-like types (Annavajhala et al., 2019; Zhiyong et al., 2021). In Spain, a study on isolates performed during 2013–2015 found that *E. cloacae* was the second most common carbapenemase-producing organism. Interestingly, carbapenem-resistance in *E. cloacae* was more frequently caused by carbapenemase-independent mechanisms, related to enzymes with low hydrolytic activity against carbapenems combined with decreased intracellular antibiotic accumulation due to porin loss or increased active efflux (Oteo et al., 2015). Similar results were obtained in a recent study in Spain including 401 CPE strains: *K. pneumoniae* (73.3%), *Enterobacter cloacae* complex (13.5%), *Escherichia coli* (4.5%), *Klebsiella oxytoca* (3.5%), *Citrobacter freundii* (2.2%), and others (2.7%; Vázquez-Ucha et al., 2021).

Carbapenem resistance mechanisms in *P. aeruginosa* are often unrelated to carbapenemase production, although this situation is quite variable in different regions around the world (Glen and Lamont, 2021; Zhiyong et al., 2021; Lepe and Martínez-Martínez, 2022). Carbapenem resistance in isolates lacking carbapenemases is a result of a combination of overproduction/structural modification of the chromosomal AmpC enzyme, loss or modification of the OprD porin, and overexpression of the MexAB-OprM efflux system (Alvarez-Ortega et al., 2011; Del Barrio-Tofiño et al., 2019; Glen and Lamont, 2021; Lepe and Martínez-Martínez, 2022). In Spain, strains producing carbapenemase are still rare, although their importance is increasing in recent years. In a national survey conducted in Spanish hospitals in 2017 (Del Barrio-Tofiño et al., 2017), considering 150 XDR isolates, 79% of them overproduced AmpC and presented an altered OprD porin, while the remaining 21% produced carbapenemases (mostly VIM and GES enzymes). The most frequent XDR high-risk clone was *P. aeruginosa* ST175 (Del Barrio-Tofiño et al., 2017). In a more recent multicenter study (51 hospitals, 1,445 isolates) rates of resistance to imipenem and meropenem were 15.6 and 14.1%, respectively, but only 2.7% of the strains produced a carbapenemase (VIM: 1.9%; GES-5: 0.5%; IMP: 0.3%; Del Barrio-Tofiño et al., 2019).

The primary objective of this study was to determine the prevalence of carbapenemase enzymes among CPE and CPPA in Spain over a five-year period (January 2014 to December 2018). The secondary objectives were to describe the demographic characteristics of the infected patients, to determine the different types and subtypes of carbapenemases and their geographic distribution in Spain, and to illustrate the evolution of carbapenemases throughout the study period.

## Materials and methods

Data from CPE and CPPA were collected in a National retrospective multicenter (30 Spanish hospitals) study, from January 2014 to December 2018.

The first isolate of carbapenemase-producing *E. coli*, *K. pneumoniae*, *E. cloacae* complex, *K. (E.) aerogenes*, or *P. aeruginosa* per patient and year cultured from clinical samples obtained for diagnosis of infection in hospitalized patients was included. Isolates obtained from surveillance samples were excluded. Bacterial identification and susceptibility testing had been performed in the participating centres using combined identification and antibiogram panels by automated commercial systems (Table 1). Carbapenemase detection and identification of the major families were determined using established phenotypic and genotypic methods (see below) in the participating centres, as shown in Table 2. Briefly, mCIM (modified carbapenem inactivation method), Hodge Test and/or Carba-NP test were used as screening methods for carbapenemase detection, and immunochromatography and/or molecular methods were the main methods for carbapenemase type identification. Information about the particular allele of carbapenemase determined by molecular methods (in house PCR or sequencing) was reported in few cases (Table 3).

**Table 1:** Susceptibility methods used for testing the indicated number (n) and percentages (%) of evaluated isolates in the participating centres.

			Diffusion method				
Commercial system			None	Disk diffusion	Gradient strips	Disk + Gradient Strips	Total
	None	*n*/%	7/0.3	108/4	2/0.1	86/3.2	203/7.5
	Microscan Walkaway	*n*/%	1414/52.3	84/3.1	123/4.5	11/0.4	1632/60.4
	Vitek	*n*/%	411/15.4	61/2.3	142/5.3	87/3.3	701/25.9
	Phoenix	*n*/%	119/4.5	0	1	0	120/4.4
	Sensititre	*n*/%	22/0.8	1	0	0	23/0.9
	Microscan Walkaway+Sensititre	*n*/%	17/0.6	0	0	0	17/0.6
	Vitek+Sensititre	*n*/%	0	0	8/0.3	0	8/0.3
Total		*n*/%	1990/73.6	254/9.4	276/10.2	184/6.8	2704/100

Microscan Walkaway (Beckman Coulter), Vitek (bioMérieux), Phoenix (BD), Sensititre (Thermo Fisher Scientific).

**Table 2:** Methods for carbapenemase identification.

Carbapenemase screening methods 500 (18.5)	Methods for carbapenemase identification 2,692 (99.5)	Molecular methods 1712 (63.6)	Immunochromatography 887 (32.9)
Type *n* (%)	Type *n* (%)	Type *n* (%)	Type *n* (%)
mCIM 409 (81.8) mHT 50 (10) Colorimetric 41 (8.2)	Molecular 1,571 (58.4) IC 804 (29.8) Sinergy-inhibitors 168 (6.2) IC + Molecular 72 (2.7) Molecular+S-I 66 (2.5) IC + Sinergy-inhibitors 8 (0.3) IC + Molecular+S-I 3 (0.1)	PCR in-house 578 (33.8) GeneXpert Cepheid 577 (33.7) OXVIKPND PCR assay (Progenie) 150 (8.8) PCR Allplex Entero DR assay (Seegene) 122 (7.1) EAZYPLEX 62 (3.6) PCR BD MAX Xheck-points CPO 50 (2.9) NanoSphere/Verigene 47 (2.7) PCR CNM-Carlos III 39 (2.3) Genie II Optigene 26 (1.5) Whole genome sequencing (Illumina) 23 (1.3) Sanger Sequencing 18 (1.1) PCR AMR (AB analitica) 12 (0.7) PCR HAIN 7 (0.4) PCR ELITECH carbas 1 (0.1)	NG Biotech (NG Test Carba-5) 381 (43) OXA-48 *K*SeT (CORIS Bioconcept) 211 (23.8) O.K.N.V.I RESIST5 (CORIS Bioconcept) 100 (11.3) LETI-TEST 89 (10) CORIS Bioconcept 66 (7.4) Resist-3 OKN *K*SeT (CORIS Bioconcept) 40 (4.5)

mCIM, Modified carbapenem inactivation method; mHT, modified Hodge Test; IC, Immunochromatography; S-I, Sinergy-inhibitors.

**Table 3:** Microorganisms and type of carbapenemase isolated (n/%) in Enterobacterales and *Pseudomonas aeruginosa*.

Enterobacterales (2,280/84.7)				*Pseudomonas aeruginosa* (412/15.3)
*Klebsiella pneumoniae* (1821/79.9)	*Enterobacter cloacae* complex (334/14.6)	*Escherichia coli* (114/5)	*Klebsiella* (*Enterobacter*) *aerogenes* (11/0.5)	
OXA-48-like (1,336/73.4) KPC (296/16.3)^a^ VIM (125/6.9)^b^ NDM (34/1.8)^c^ OXA-48-like+NDM (24/1.3) OXA-48-like+VIM (1/0.05) KPC + VIM (1/0.05) IMP (1/0.05) GES-6 (1/0.05) Other MBL (2/0.1)	VIM (125/37.4)^d^ OXA-48-like (75/22.4) NDM (64/19.2)^e^ GES (44/13.2)^f^ KPC (13/3.9) IMP (11/3.3) OXA-48-like+KPC (1/0.3) OXA-48-like+VIM (1/0.3)	OXA-48-like (82/71.9) VIM (17/14.9)^g^ NDM (7/6.1) GES (3/2.6) KPC (2/1.8) KPC + VIM (1/0.9) OXA-48-like+NDM (1/0.9) Other MBL (1/0.9)	VIM (4/36.3) OXA-48-like (2/18.2) KPC (2/18.2) IMP (1/9.1) NDM (1/9.1) Other MBL (1/9.1)	VIM (365/88.6) ^h, i^ IMP (10/2.4) ^j^ OXA-48-like (3/0.7) Other MBL (34/8.3)

Information about the particular allele of the carbapenemase was available in 254 (9.4%) cases as follows: ^a^KPC-3 (100/33.8); ^b^VIM-1 (21/16.8); ^c^NDM-1 (2/5.8); ^d^VIM-1 (44/35.2); ^e^NDM-5 (1/1.5); ^f^GES-6 (44/100); ^g^VIM-1 (3/17.6); ^h^VIM-1 (21/5.7); ^i^VIM-2 (19/5.2); ^j^IMP-1 (1/10).

A structured questionnaire was completed (details presented in Supplementary Table S1), which included general questions (study site identification, susceptibility testing methodology, use of automated antibiogram methods, susceptibility criteria (EUCAST or CLSI) for definition of clinical categories, methods for carbapenemase detection) and questions related to the included patients/microorganisms (demographic data, sample type, service of hospitalization, bacterial species, type of carbapenemase and, when available, specific carbapenemase allele) All data were registered by researchers using the REDCap platform.

The results were analyzed using IBM SPSS Statistics 29.0.0. Age is expressed as average, median and range. The results are expressed as number of cases (n) and percentage (%) in all the variables.

The study was classified by the Spanish Agency of Drugs and Pharmaceutical Products (AEMPS as in Spanish) as a “Post authorization Study with Other designs different to the Prospective follow-up” (EPA-OD, as Spanish translation) with protocol code MSD-CAR-2020-01. Based on the classification of Post authorization Study, and following the Spanish legislation, “Orden SAS 2470/2009.” The study was reviewed and approved by the Hospital Universitario Reina Sofía Ethics Committee on 29 July 2020.

## Results

A total of 2,704 carbapenemase-producing microorganisms were included, although in 12 (0.4%) cases only carbapenemase production was performed, without determination of the type of carbapenemase. Accordingly, the type of carbapenemase was identified in 2.280 CPE (84.7%) and 412 CPPA (15.3%; Table 3). In all, 1,678 cases (62.1%) were men and 1.026 (37.9%) were women, with an average age of 68.2 years (range 0–115 years). Patients were hospitalized in medical, surgical or intensive care units in 1.503 (55.6%), 720 (26.7%), or 481 (17.8%) cases, respectively. A total of 1.064 (39.3%) organisms were cultured from urine samples, followed by respiratory samples (502, 18.6%), blood (451, 16.8%), intraabdominal samples (201, 7.4%) and other samples, as detailed in Supplementary Table S2.

Different phenotypic and genotypic methods were used in the participating centres for detecting and identifying carbapenemase types, as shown in Table 2. Phenotypic screening assays (including mCIM test, modified Hodge test or colorimetric methods) were used in 500 (18.5%) cases. As previously indicated, in 12 (0,4%) cases only a screening method was performed for carbapenemase detection. This was the case for 1 *E. coli*, 8 *K. pneumoniae*, 1 *K. aerogenes*, and 2 *P. aeruginosa*. The most frequent techniques used for identifying the major families of carbapenemases were molecular methods, in 1.712 (63.6%) cases, followed by immunochromatography in 887 (32.9%) cases, alone or in combination with different methods as synergy-inhibitors. Between 2014–2017, molecular techniques were the most frequent methods used (63.7–73.2%), but in 2018 immunochromatography methods were used more frequently compared to molecular methods (53.3 vs. 47.4%).

The distribution of carbapenemase types by bacterial species is shown in Table 3. Globally, the most frequent types of carbapenemases in Enterobacterales were OXA-48-like, either alone or in combination with other enzymes (1.523 cases, 66.8%) and VIM (365 cases, 88.6%) in *P. aeruginosa*.

Among Enterobacterales, carbapenemase-producing *K. pneumoniae* was reported in 1.821 cases (79.9%), followed by *E. cloacae* complex in 334 cases (14.6%). The most frequent types of carbapenemases found in *K. pneumoniae*, were OXA-48-like (as a single enzyme in 73.5% of cases and combined with NDM or VIM in 1.3 and 0.05% of cases, respectively), followed by KPC (alone, 16.3%, or combined with VIM, 0.05%).

**Figure 1:**
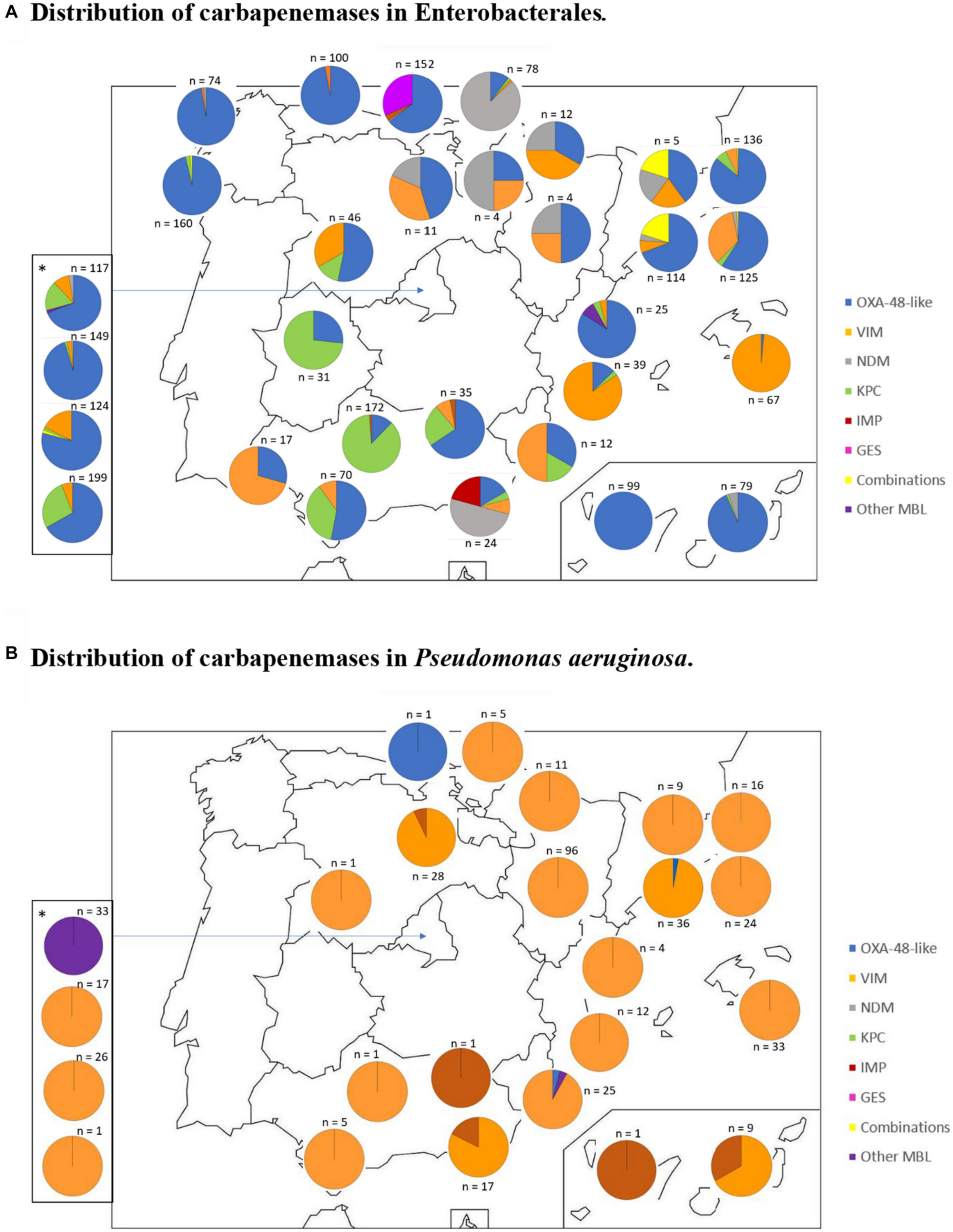
Geographical distribution of carbapenemase-producing microorganisms. Distribution of carbapenemases in Enterobacterales. Distribution of carbapenemases in *Pseudomonas aeruginosa*. The map shows a pie chart at each participating hospital with the distribution and total number of carbapenemases in Enterobacterales **(A)** and *Pseudomonas aeruginosa* **(B)**. *Pie charts corresponding to the Community of Madrid participating hospitals are shown at the side of the map in order to avoid overlaping.

The geographical distribution of carbapenemase types is presented in Figures 1A,B. In Enterobacterales, KPC is mainly present in the South and Southeast of Spain, while OXA-48-like is prevalent in the rest of the country. Regarding *P. aeruginosa*, VIM is widely distributed throughout the country.

**Figure 2:**
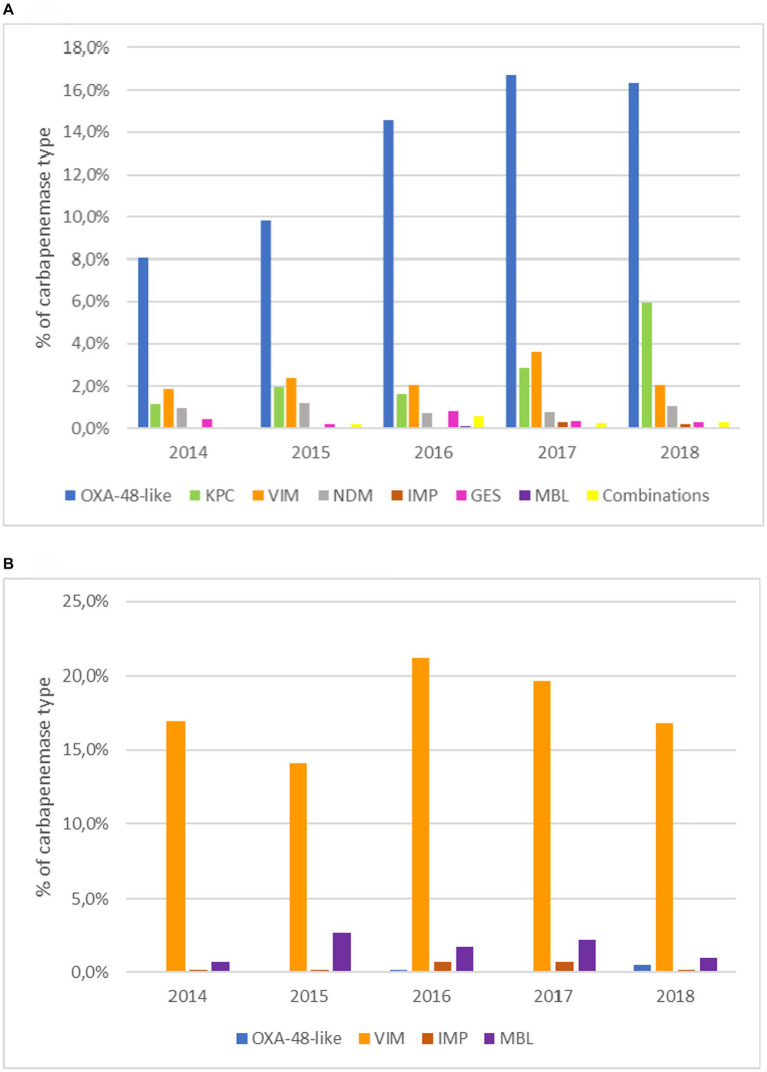
Temporal evolution of carbapenemase types. **(A)** In Enterobacterales. **(B)** In *Pseudomonas aeruginosa*. MBL: metallo-β-lactamases; Combination of different carbapenemases (OXA-48-like+KPC, OXA-48-like+NDM, OXA-48-like+VIM, KPC + VIM).

The temporal distribution of carbapenemase types is presented in Figure 2. An increasing number of organisms producing OXA-48-like enzymes was observed from 2014 to 2017. Organisms producing KPC enzymes were more frequent in 2017–2018 compared to 2014–2016.

Table 1 shows the distribution of methods used by the participating centres for antimicrobial susceptibility testing. In most cases (2.501, 92.5%), antimicrobial susceptibility testing of the corresponding organism was performed using commercial broth microdilution panels. Diffusion methods were the only susceptibility testing assays performed in 196 (7.2%) cases. The MicroScan and Vitek systems were used in 1.649 cases (61%) and 709 cases (26.2%), respectively. Either disk-diffusion or gradient-strips diffusion assays were performed in 146 (5.4%) and 274 (10.1%) cases, respectively, and both assays were performed simultaneously in an additional 98 (3.6%) cases, additionally to commercial broth microdilution panels.

Clinical categories were most often defined following EUCAST recommendations (78.9% of cases), considering the criteria established at the time when the corresponding organism was evaluated. Clinical categories of tested antimicrobial agents against the different microorganisms are presented in Table 4. For Enterobacterales, more than 90% of all isolates were resistant to piperacillin-tazobactam, cefotaxime and ceftazidime. Resistance to ertapenem (85.4%) was higher than to meropenem and imipenem (47.3 and 41.5%, respectively). In the case of *P. aeruginosa*, the highest percentages of resistance (excluding cefotaxime and ertapenem) were observed for cefepime (90.8%), ceftazidime (90.5%), meropenem (89.7%), ciprofloxacin (86.3%) and piperacillin-tazobactam (85.1%) and imipenem (81.0%).

**Table 4:** Clinical categories of antimicrobial agents reported by the participating centres by family of carbapenemase-producing microorganisms.

	Enterobacterales			*P. aeruginosa*		
	% R	% I	% S	% R	% I	% S
Piperacillin-tazobactam	96.7%	1.7%	1.6%	85.1%	7.8%	7.1%
Cefotaxime	93.5%	1.2%	5.3%	--	--	--
Ceftazidime	91.3%	0.8%	7.9%	90.5%	4.9%	4.6%
Cefepime	87.3%	4.5%	8.2%	90.8%	5.2%	4%
Aztreonam	76.6%	9.4%	14%	29.9%	43.1%	27%
Ertapenem	85.4%	10.4%	4.2%	--	--	--
Imipenem	41.5%	20.3%	38.2%	81.0%	10.4%	8.6%
Meropenem	47.3%	14.4%	38.3%	89.7%	8.2%	2.1%
Gentamicin	59.3%	3.5%	37.2%	74.6%	8.2%	17.2%
Amikacin	23.1%	6.9%	70%	58.2%	9.7%	32.1%
Ciprofloxacin	89.5%	2%	8.5%	86.3%	2.4%	11.3%
Colistin	12.7%	0.2%	87.1%	5.4%	0.3%	94.3%

The percentages of resistance to different antimicrobials by type of carbapenemase are shown in Table 5. For Enterobacterales, resistance rates to carbapenems varied considerably depending on the specific type of carbapenemases, but it was in practically all cases higher for ertapenem. The lowest percentages of resistance of the tested compounds were noted for the non-β-lactam colistin and amikacin, both in Enterobacterales and in *P. aeruginosa*. Resistance rates to imipenem and meropenem were particularly low for GES-producing isolates. Interestingly, resistance to aztreonam in isolates producing NDM was as high as 93.9%, with values of 49.2% for VIM producers and 33.3% for IMP producers.

**Table 5:** Resistance (%) to antimicrobial agents by carbapenemase type.

Enterobacterales (*n*)	OXA-48-like (1495)	KPC (313)	VIM (271)	NDM (106)	IMP (13)	GES (48)	Other MBL (4)	Others^*^ (30)
Piperacillin-tazobactam	96.7%	95.3%	98.7%	100%	58.3%	94.7%	100%	100%
Cefotaxime	90.8%	99.6%	99.6%	97%	92.3%	95.8%	100%	100%
Ceftazidime	88.2%	95.8%	99.2%	97%	100%	97.9%	100%	96.7%
Cefepime	84.3%	93.2%	96%	96.5%	75%	75%	100%	96.6%
Aztreonam	83.1%	71.5%	49.2%	93.9%	33.3%	NT	50%	100%
Ertapenem	85.4%	98.8%	73.1%	95.8%	61.5%	52.1%	NT	100%
Imipenem	31%	76.8%	41.8%	87.3%	30.8%	2.1%	100%	90%
Meropenem	39.2%	84.9%	39.2%	93.5%	38.5%	2.6%	100%	100%
Gentamicin	58.3%	73.7%	55.5%	28.4%	53.8%	70.8%	100%	93.3%
Amikacin	17.9%	43.6%	11.8%	62.2%	0%	0%	25%	72%
Ciprofloxacin	91.6%	92.2%	72.8%	96.2%	76.9%	83.3%	100%	100%
Colistin	11.1%	13.7%	10.8%	35%	0%	0%	0%	16.7%

*P. aeruginosa* (*n*)	OXA-48-like (3)	VIM (365)	IMP (10)	Other MBL (34)
Piperacilin-tazobactam	100%	86.7%	30%	82.4%
Ceftazidime	66.7%	90.6%	100%	88.2%
Cefepime	100%	90.1%	100%	94.1%
Aztreonam	50%	31.9%	11.1%	12.1%
Imipenem	33.3%	79.3%	100%	97%
Meropenem	100%	89.1%	100%	90.9%
Gentamicin	100%	73.1%	90%	88.2%
Amikacin	66.7%	57.8%	77.8%	58.8%
Ciprofloxacin	66.7%	87.5%	50%	85.3%
Colistin	0%	5.5%	0%	0%

* Others: combination of different carbapenemases (OXA-48-like + KPC, OXA-48-like + NDM, OXA-48-like + VIM, KPC + VIM).

## Discussion

This study represents an effort to evaluate the frequency of the different carbapenemase enzymes among CPE (including not only *K. pneumoniae* and *E. coli*, but also *K. aerogenes* and *E. cloacae* complex) and CPPA over an extended period and in a large number of hospitals in Spain. Previous studies have focused on organisms isolated during a brief period or concentrated on a limited number of species (Miro et al., 2013; Oteo et al., 2015; Del Barrio-Tofiño et al., 2017; Grundmann et al., 2017; Del Barrio-Tofiño et al., 2019; Horcajada et al., 2019; Cañada-García et al., 2022).

Most Spanish laboratories have implemented methodologies for carbapenemase identification, at least to family/type level. The most frequently used methods were commercial PCR or in-house PCR assays between 2014 and 2017 (63.7–73.2%). In many cases, these were supplemented with immunochromatographic detection of major carbapenemase families and phenotypic/colorimetric tests. Different multiplex PCR commercial system were used during the study period including genes of the most frequent carbapenemases (e.g., KPC, VIM, IMP, NDM…). These systems are time-saving, have high levels of sensitivity and specificity but are quite expensive and miss to identify less frequent carbapenemases (e.g., GES, IMI…) or specific alleles (e.g., IMP-19 or IMP-66 in GenXpert system; Cui et al., 2019; Kanahashi et al., 2021). It should be noted that in the present study the use of in-house PCR and whole genome sequencing methodology has increased during 2018 (7%) compared to the previous years. Actually, this methodology is useful to report information about specific alleles and epidemiological information. However, it is not available in all microbiology laboratories, which explains the increased use of methods based on immunochromatography. In the present study, despite the fact that molecular methods have been used more frequently during the 5 years, the use of immunochromatography has increased over the years of the study (18.6–53.3%) because of price, reliability and easy-to-perform method, as opposed to molecular methods. These approaches are essentials not only for a more effective control of carbapenemase-producing organisms but also for guiding appropriate therapy of patients infected with these bacteria, especially considering the availability of new agents (particularly new β-lactam/β-lactamase inhibitor combinations) which have significant differences in their spectrum of activity against CPE and CPPA.

MIC values of clinically relevant antimicrobial agents are usually determined in our country using commercial semi-automatic methods, particularly MicroScan WalkAway (Beckman Coulter) and to a lesser extent, Vitek (bioMérieux; Table 1). A minority of laboratories used only the disk-diffusion assay, either alone or combined with gradient strips, when testing CPE and CPPA. The clinical categories of the tested compounds are largely defined using EUCAST criteria, which is likely influenced by the multiple activities and reports from the Spanish national antimicrobial susceptibility testing committee (CoEsAnt, website: http://coesant-seimc.org; Cantón et al., 2020; Larrosa et al., 2020, 2022).

The results from this study indicate that the most frequent carbapenemases in Enterobacterales in Spain over the study period are OXA-48-like and KPC, which is consistent with previous studies conducted in recent years (Miro et al., 2013; Oteo et al., 2015; Grundmann et al., 2017; Cañada-García et al., 2022). The most common species among CPE have been *K. pneumoniae* and *E. cloacae* complex, with OXA-48-like enzymes being the most frequent carbapenemases in *K. pneumoniae* (also observed in *E. coli*). In *E. cloacae* complex, VIM-type enzymes have been the most frequent. VIM enzymes have also been the most common carbapenemases in *P. aeruginosa*, which is consistent with the results of previous studies in Spain (Del Barrio-Tofiño et al., 2017, 2019; Horcajada et al., 2019).

It is important to note that this study has focused on carbapenemase-producing organisms rather than carbapenem-resistant gram-negative bacteria. Recent reports from our country (Miro et al., 2013; Oteo et al., 2015; Del Barrio-Tofiño et al., 2017, 2019; Grundmann et al., 2017; Horcajada et al., 2019; Cañada-García et al., 2022) have already demonstrated that while carbapenem resistance in *K. pneumoniae* and *E. coli* is mainly related to carbapenemase production, carbapenem resistance in *P. aeruginosa* and *E. cloacae* complex is usually related to the production of β-lactamases with a low/moderate hydrolytic efficiency of carbapenems, combined with reduced intrabacterial drug accumulation due to porin loss and/or overexpression of efflux pumps (Del Barrio-Tofiño et al., 2017, 2019; Horcajada et al., 2019).

In addition to OXA-48-like enzymes in Enterobacterales and VIM carbapenemases in *E. cloacae* complex and *P. aeruginosa*, KPC has also been quite common among *K. pneumoniae* isolates, while NDM, IMP and GES have been found less frequently. The recently published CARB-ES-19 study (Cañada-García et al., 2022) has documented that the main KPC allele disseminated in Spain is KPC-3, but other studies have also identified new KPC (Hernández-García et al., 2021, 2022; Guzmán-Puche et al., 2022) responsible for resistance to ceftazidime-avibactam which, in some cases, cause collateral susceptibility to carbapenems.

From 2014 to 2017, there has been a continuous increase in the identification of isolates (mostly *K. pneumoniae*) producing OXA-48-like enzymes, although the number of isolates with this type of enzymes stabilized in 2018. In contrast, the number of KPC-producing organisms (in all cases Enterobacterales, as this enzyme was not identified in *P. aeruginosa* in our study), increased in 2017 and particularly in 2018 when it became the second most frequent β-lactamase in Spain, as also observed in the CARB-ES-19 study which included carbapenemase-producing *K. pneumoniae* and *E. coli* (Cañada-García et al., 2022).

An analysis of the geographical distribution of the most frequent carbapenemases in Spain indicates that in Enterobacterales, KPC seems to be more prevalent in the Southern and South-eastern regions of the country, while OXA-48-like is more prevalent in the remaining regions. These data contrast with the findings of a 2015 report (with organisms collected between February and May 2013; Oteo et al., 2015) which indicated that many provinces were free from OXA-48-like producing Enterobacterales, and KPC producers were identified in just two central provinces and in one Eastern province. For *P. aeruginosa*, VIM is widely distributed all over the country.

In the CPE cases, OXA-48-like strains presented high resistance level to piperacillin-tazobactam and cephalosporins (cefotaxime, ceftazidime and cefepime) suggesting those strains are associated to ESBL production. Interestingly, resistance to aztreonam in CPE was between 33 and 50% except for NDM which was over 93%. On the other hand, in CPPA resistance to aztreonam was under 32% but in IMP and other metallo-β-lactamases was under 12%. Therefore, identification of metallo-carbapenemase type prior to antimicrobial susceptibility testing information is available can be useful for empiric treatment in a high proportion of cases. Resistance to colistin was under 17% in all CPE and under 5.5% in all types of metallo-β-lactamases in CPPA thus being a therapeutic option. GES producers showed quite low levels of resistance to imipenem or meropenem according to their low hydrolytic activity, but >50% resistance to ertapenem so it should be included in susceptibility testing methods.

This study has several limitations, most of which are inherent to its retrospective design. While we have identified the carbapenemase families of major epidemiological and clinical relevance, in most cases the specific alleles have not been defined, as sequencing data were not available from the participating centers. MICs of carbapenems and other agents were determined in most centres using commercial panels, which, in many cases, do not include the necessary number of dilutions to allow precise MIC definition or the correct application of EUCAST clinical breakpoints. We suggest that manufacturers follow the CoEsAnt recommendations (Zhiyong et al., 2021) for the ideal selection of agents to be used in panels for automated systems. The use of commercial panels also resulted in a lack of information on new agents with activity against carbapenemase producing organisms (such as ceftazidime-avibactam, meropenem-vaborbactam, imipenem-relebactam, cefiderocol, eravacycline, etc.) since these panels did not include the indicated drugs. A new study considering these new therapeutic options is warranted. Finally, only the most frequent CPE species have been considered, and no information on organisms of genera *Citrobacter*, *Proteus*, *Providencia*, *Morganella*, *Hafnia*, etc. was obtained. Similarly, our study only considered *P. aeruginosa*, although some reports also indicate the importance of other species, particularly *P. putida* and related species, as carbapenemase producers (Gilarranz et al., 2013; Molina et al., 2014; Ocampo-Sosa et al., 2015). There is also a limitation in detecting less prevalent carbapenemases as GES enzymes by immunochromatographic or PCR assays. In Spain, GES enzymes have been described in *P. aeruginosa* in some reports (Herrera-Espejo et al., 2022; Recio et al., 2022), but in this study maybe less represented because of assays limitations.

The information from this study help to understand the situation and evolution of the main species of carbapenemase-producing Enterobacterales and *P. aeruginosa* in Spain, with practical implications for control and optimal treatment of infections caused by these MDR organisms. Continuous efforts should be maintained in the future in this area, with a focus on maintaining the clinical efficacy of recently introduced new therapeutic agents.

## Data availability statement

The raw data supporting the conclusions of this article will be made available by the authors, without undue reservation.

## Ethics statement

The studies involving humans were approved by Comité de ética de la investigación con medicamentos provincial de Córdoba. The studies were conducted in accordance with the local legislation and institutional requirements. Written informed consent for participation was not required from the participants or the participants’ legal guardians/next of kin in accordance with the national legislation and institutional requirements.

## Author contributions

JD-R, IG-A, and LM-M designed and coordinated the study. IG-A made statistical analysis. IG-A and LM-M made the analysis of the results and wrote the manuscript. All centres’ collaborators selected cases to be included and performed critical analysis of the obtained results and discussion. All authors contributed to the article and approved the submitted version.

## Funding

This study is sponsored by MSD Spain. IG-A, XM, PM, JC-M, RC, NL-E, and LM-M are supported by CIBER de Enfermedades Infecciosas (CIBERINFEC), Instituto de Salud Carlos III, Madrid, Spain. AD-I, PM, and JC are supported by CIBER de Enfermedades Respiratorias (CIBERes), Instituto de Salud Carlos III, Madrid, Spain.

## Conflict of interest

LM-M has been a consultant for MSD, Shionogi and Fastinov, has served as speaker for Merck, Astra-Zeneca, Astellas, and Becton Dickinson and has received research support from MSD, Shionogi, Janssen-Cilag and Pfizer. JD-R is an employee of MSD Spain. RC has participated in educational programs organized by MSD, Pfizer and Shionogi and has received research support form MSD, and Venatrox. NL-E has been a consultant for MSD, Menarini, Shionogi and Fastinov and has served as a speaker for MSD, Pfizer, Menarini, Shionogi, Biomerieux and Accelerate Diagnostics.

The remaining authors declare that the research was conducted in the absence of any commercial or financial relationships that could be construed as a potential conflict of interest.

## Publisher’s note

All claims expressed in this article are solely those of the authors and do not necessarily represent those of their affiliated organizations, or those of the publisher, the editors and the reviewers. Any product that may be evaluated in this article, or claim that may be made by its manufacturer, is not guaranteed or endorsed by the publisher.
