# Supplementary figures and images for: The CARBA-MAP study: national mapping of carbapenemases in Spain (2014–2018)
Source: Front Microbiol. 2023 Sep 8;14:1247804. doi: 10.3389/fmicb.2023.1247804 (PMC10516297; doi:10.3389/fmicb.2023.1247804)

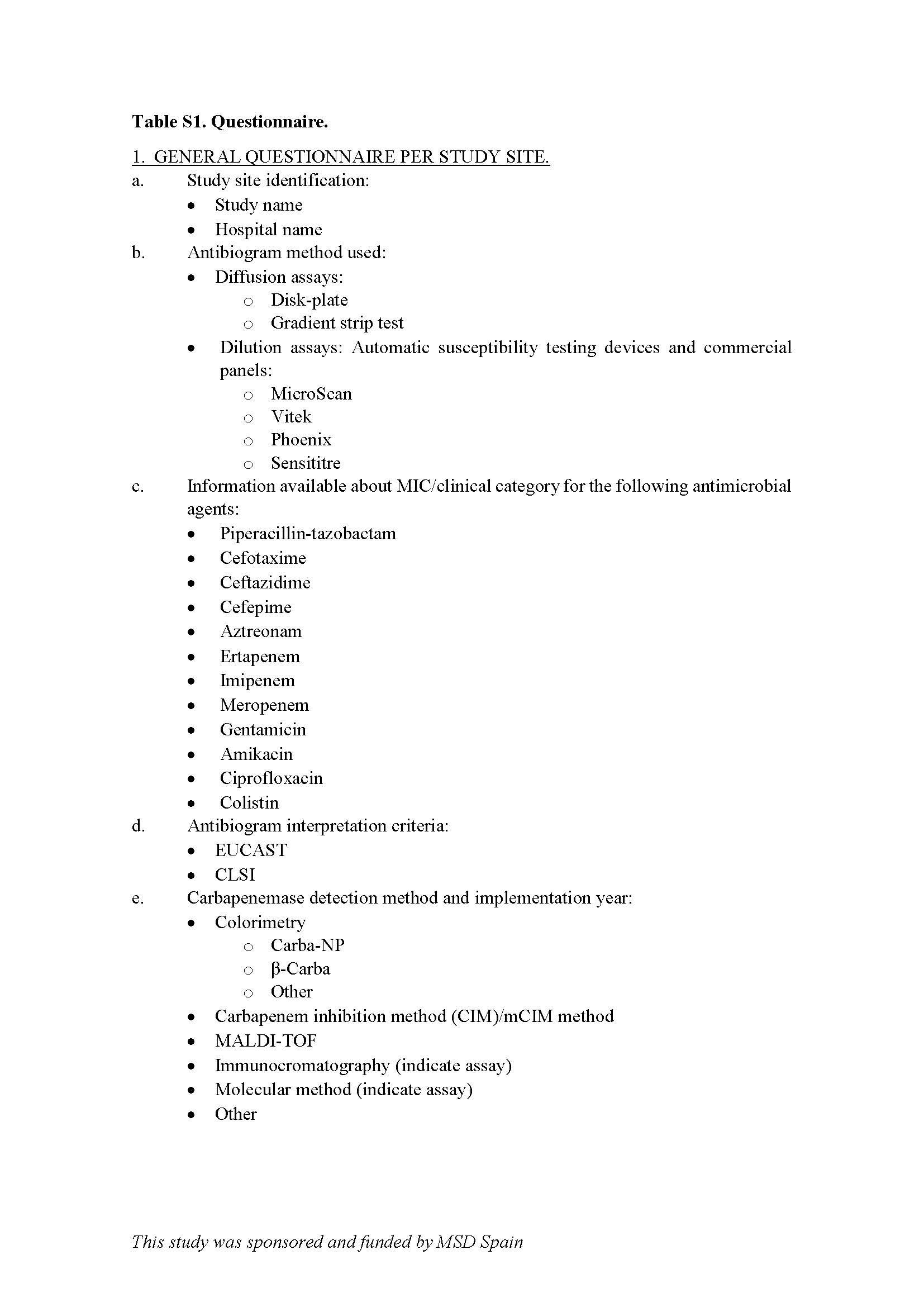

Supplement: Supplementary file 1 [file Image_1.JPEG]

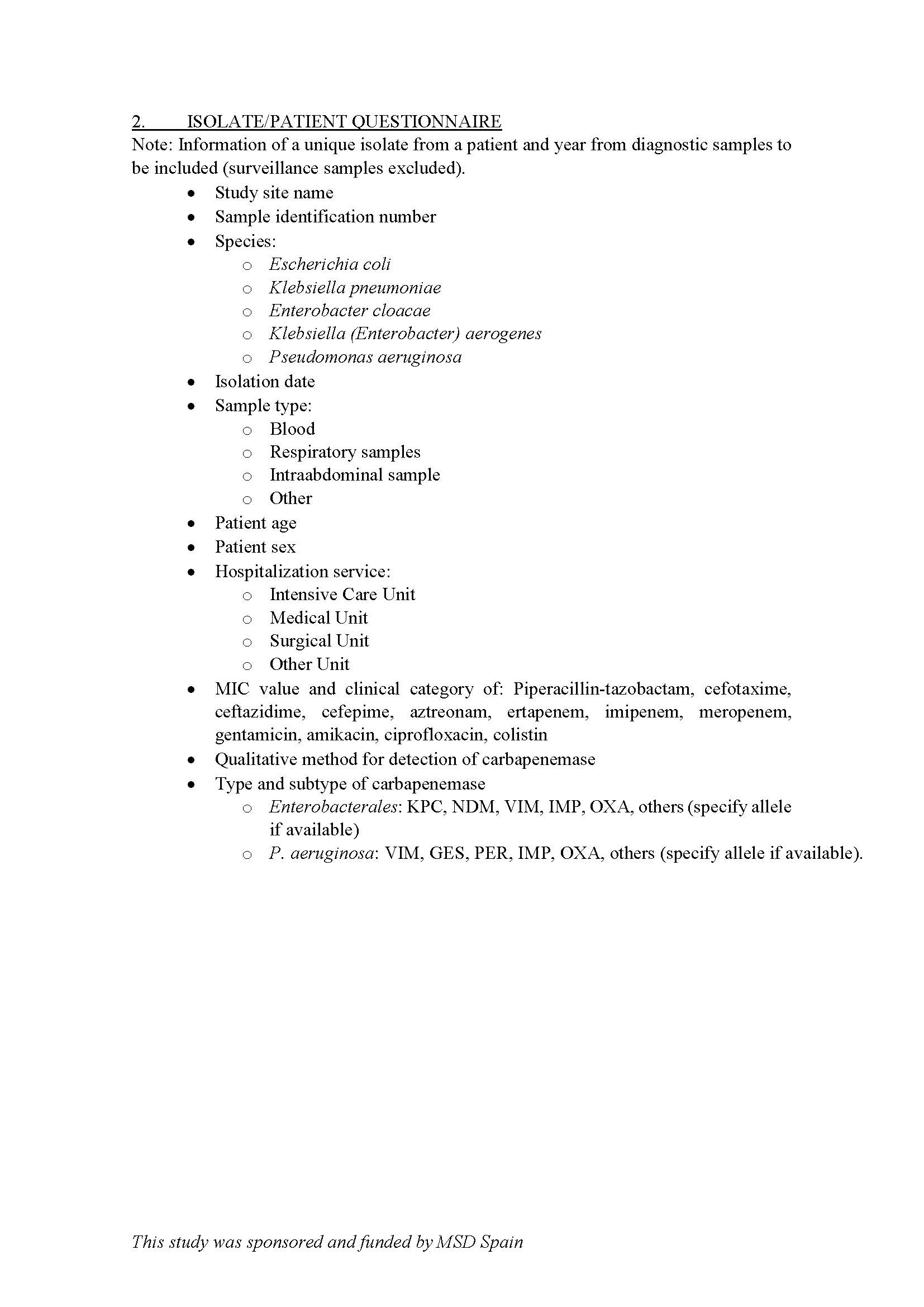

Supplement: Supplementary file 2 [file Image_2.JPEG]

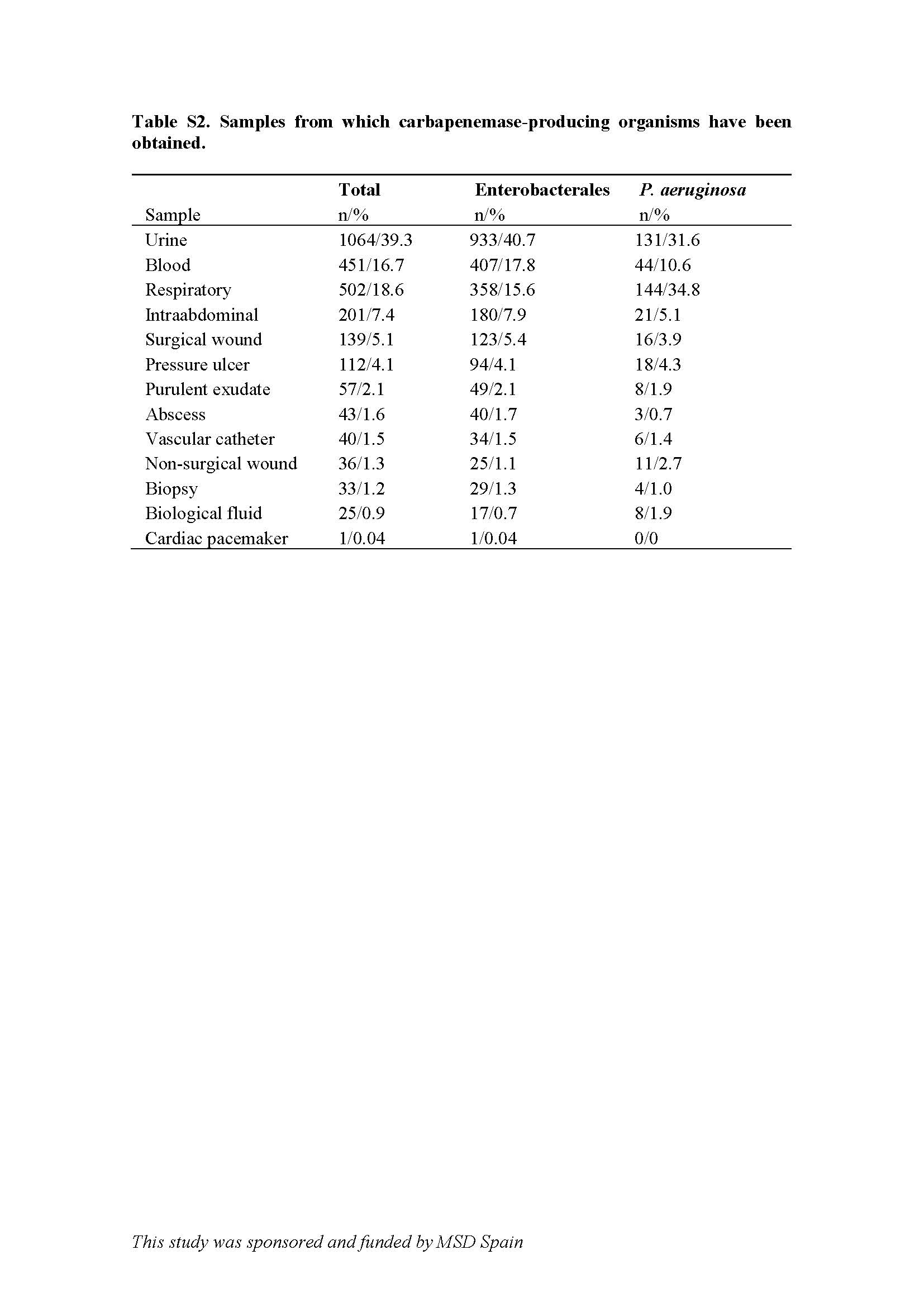

Supplement: Supplementary file 3 [file Image_3.JPEG]
